# Synergistic use of plant-prokaryote comparative genomics for functional annotations

**DOI:** 10.1186/1471-2164-12-S1-S2

**Published:** 2011-06-15

**Authors:** Svetlana Gerdes, Basma El Yacoubi, Marc Bailly, Ian K Blaby, Crysten E Blaby-Haas, Linda Jeanguenin, Aurora Lara-Núñez, Anne Pribat, Jeffrey C Waller, Andreas Wilke, Ross Overbeek, Andrew D Hanson, Valérie de Crécy-Lagard

**Affiliations:** 1 Fellowship for Interpretation of Genomes, Burr Ridge, IL, USA; 2 Department of Microbiology and Cell Science, University of Florida, Gainesville, FL, USA; 3 Department of Horticultural Sciences, University of Florida, Gainesville, FL, USA; 4 Computation Institute, University of Chicago, Chicago, IL, USA

## Abstract

**Background:**

Identifying functions for all gene products in all sequenced organisms is a central challenge of the post-genomic era. However, at least 30-50% of the proteins encoded by any given genome are of unknown or vaguely known function, and a large number are wrongly annotated. Many of these ‘unknown’ proteins are common to prokaryotes and plants. We set out to predict and experimentally test the functions of such proteins. Our approach to functional prediction integrates comparative genomics based mainly on microbial genomes with functional genomic data from model microorganisms and post-genomic data from plants. This approach bridges the gap between automated homology-based annotations and the classical gene discovery efforts of experimentalists, and is more powerful than purely computational approaches to identifying gene-function associations.

**Results:**

Among Arabidopsis genes, we focused on those (2,325 in total) that (i) are unique or belong to families with no more than three members, (ii) occur in prokaryotes, and (iii) have unknown or poorly known functions. Computer-assisted selection of promising targets for deeper analysis was based on homology-independent characteristics associated in the SEED database with the prokaryotic members of each family. In-depth comparative genomic analysis was performed for 360 top candidate families. From this pool, 78 families were connected to general areas of metabolism and, of these families, specific functional predictions were made for 41. Twenty-one predicted functions have been experimentally tested or are currently under investigation by our group in at least one prokaryotic organism (nine of them have been validated, four invalidated, and eight are in progress). Ten additional predictions have been independently validated by other groups. Discovering the function of very widespread but hitherto enigmatic proteins such as the YrdC or YgfZ families illustrates the power of our approach.

**Conclusions:**

Our approach correctly predicted functions for 19 uncharacterized protein families from plants and prokaryotes; none of these functions had previously been correctly predicted by computational methods. The resulting annotations could be propagated with confidence to over six thousand homologous proteins encoded in over 900 bacterial, archaeal, and eukaryotic genomes currently available in public databases.

## Background

Accurate characterization of as many genes as possible is a central challenge of the post-genomic era and an essential precondition for progress in systems biology [1]. But this characterization is very far from completion. By various estimates, at least 30-50% of the genes of any given organism are of unknown function [2], incorrectly annotated [3,4], or have only a generic annotation such as ‘ATPase’ [5]. This problem is particularly acute for eukaryotic genomes, which are in general less well annotated than prokaryotic ones [6,7].

Moreover, with more than 6,000 genomes now (August 2010) in the pipeline, 1,354 of them eukaryotic (http://www.genomesonline.org), the numbers of unknown genes continue to increase [8] and annotation errors continue to increase even faster [9]. For some gene families up to 60% of the annotations are wrong [9]. Without specific functional annotation efforts, present and future genome information will become ever more corrupt and hard to analyze, and will thus be greatly underexploited.

The first step in linking gene to function is to define what constitutes a function, and this is not trivial. Full definition of a protein’s function requires a combination of two features or ‘dimensions’: (i) a molecular function (e.g. an enzymatic activity) and (ii) a functional context (e.g. a pathway) comprising other proteins involved in the same process. Currently most annotations in public archives convey only molecular functions, mainly assigned by homology. However, when an enzymatic activity has been annotated in this way, it may well be wrong if other genes of the same pathway are not in the genome [10]. To decide whether a protein has a truly known function, it is therefore essential to take into account both the molecular and functional context dimensions. Most automated annotation platforms use only the molecular function, but when metabolic reconstruction (i.e. pathway context) is included in the annotation process this greatly improves annotation quality [11-13].

**Figure 1:**
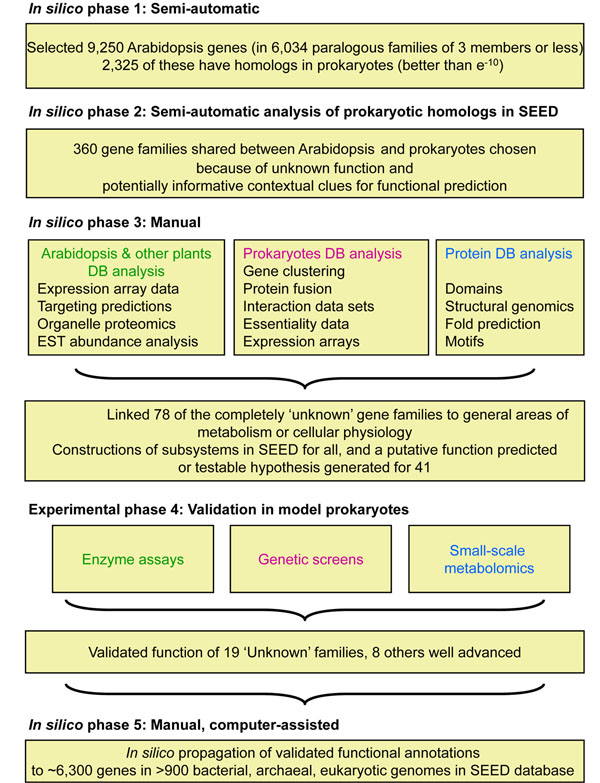
**Project workflow.** The overall strategy that combined *in silico* and experimental validation is presented showing the number of genes that were analyzed at each stage.

We and others have previously emphasized the power of cross-kingdom comparative genomics approaches to link gene and function [8,14]. This strategy was applied in the work presented here to families of unknown function shared by *Arabidopsis thaliana* and prokaryotes. Using the series of sieves summarized in Fig. 1, we combined comparative genomic and experimental validation approaches to discover the function of ‘unknowns’. Throughout this work, our primary comparative genomics platform was the SEED database and its tools [10]; the SEED is publicly available at http://www.theseed.org/Papers/20101120/.

## Results and discussion

### Selecting candidate hypothetical genes families conserved in plants and prokaryotes

#### Generation of the starting Arabidopsis gene set

The full set of 26,207 Arabidopsis genes was extracted from the re-annotated genome [15]. To predict functions for ‘unknown’ genes conserved among prokaryotes and plants, it is important to avoid large gene families because their members often have different functions. The Tribe [16] and TIGR [17] algorithms were therefore used to filter out genes belonging to families having four or more members in Arabidopsis, leaving 9,250 genes corresponding to 6,034 gene families (Table 1; personal communication, Dr. Brian Haas, The Institute for Genomic Research).

**Table 1:** Selection of candidate hypothetical genes families conserved in Arabidopsis (AT) and prokaryotes for *in silico* functional predictions and potential experimental verification – an overview.

AT gene families in this study	AT genes (and families) screened	AT genes with prokaryote homolog(s)	AT genes selected for *in silico* analysis	Gene families connected to metabolic areas	Families with specific hypotheses formulated	Families experimentally tested in this study	Families with validated functions -	
							
							in this study	by others
Singletons	3,625	666 (18.4%)	178	42	21	10	3	5
Duplets	3,204 (1,602 x2)	909 (28.4%)	190	21	13	7	3	2
Triplets ^a^	2,421 (807 x3)	849 (35.0%)	262	14	6	3	2	3
					(+1)^b^	(+1)^b^	(+1)^b^	
**Total**	**9,250 (6,034)**	**2,325 (25.1%)**	**630**	**78**	**41**	**21**^c^	**9**	**10**

^a^ Includes several Arabidopsis gene families with 4 or more paralogs

^b^ One candidate was not in the Arabidopsis set (number 4 in Table 2).

^c^ Includes 9 families with functions experimentally validated, 4 invalidated, and 8 for which experimental validation is currently in progress (see Table 2 and Table 3 for details).

#### Selecting gene families conserved between plants and prokaryotes

A second filter was applied to the 9,250 genes to retain those whose products have prokaryotic homologs. BLASTP [18] searches were performed in summer 2008 against the approximately 650 complete or almost complete microbial genomes then available in the SEED database. The probability threshold (E value) of better than 10^-10^ was imposed to ensure sufficient functional conservation between amino acid sequences included in the analysis [19,20]. Approximately one quarter of the 9,250 Arabidopsis genes tested were found to be similar to at least one prokaryotic gene (2,325 total, Supplemental Tables 1A, 1B, and 1C). Prokaryotic homologs for additional Arabidopsis genes would most probably be detected were this comparison to be repeated with the more numerous (~1,000) and more diverse microbial genomes now available.

#### Selecting hypothetical Arabidopsis/prokaryotic gene families

Several strategies were combined to extract from the set of 2,325 conserved Arabidopsis genes those whose functions are unknown or poorly known. The following sources of evidence (available as of summer 2008) were considered: (i) Arabidopsis gene annotations in the TAIR database [21]; (ii) SEED annotations of prokaryotic orthologs of Arabidopsis genes; (iii) the list of Arabidopsis proteins of unknown function (PUFs) [22] (http://bioweb.ucr.edu/scripts/unknownsDisplay.pl); and (iv) publications in PubMed and TAIR databases (or the absence thereof).

We relied mainly on the second of these sources, i.e. SEED annotations of prokaryotic orthologs of the candidate Arabidopsis genes. The nontrivial task of establishing gene orthology is greatly aided in SEED by the subsystem-based organization of annotations (described in Methods; [10,23]). We considered Arabidopsis genes to be ‘known’ and excluded them from further analysis if they or any of their prokaryotic orthologs were associated with SEED subsystems that are classified as non-hypothetical; i.e. encoding established metabolic pathways, physiological processes, or structural complexes (as opposed to experimental or hypothetical subsystems that group uncharacterized genes based on various criteria, including co-localization, co-regulation, common phenotype, etc.)

The list of Arabidopsis PUFs [22] served as a secondary resource. Three distinct PUF identification methods, complementary to our approach, have been used to compile this list: (i) BLASTP searches against proteins of known function in Swiss-Prot; (ii) Hidden Markov Model-derived searches against the Pfam domain database; and (iii) retrieval of the 'unknown' annotations from the Gene Ontology system [22]. Finally, to keep abreast of discoveries of functions for Arabidopsis genes, publications associated with selected Arabidopsis genes were extracted from the TAIR database and PubMed repeatedly during the course of this project.

This analysis yielded a set of 630 hypothetical Arabidopsis genes, corresponding to about 360 gene families common to Arabidopsis and prokaryotes that were highly enriched in those specifying proteins of unknown function. This gene set was then prioritized for in-depth *in silico* analysis as described below.

### Prioritizing Arabidopsis/prokaryotic gene families for detailed in silico analysis

#### General strategy

As the pool of 360 gene families was still too large for the labor-intensive process of in-depth comparative genomic analysis, we prioritized the candidates for further analysis based on several characteristics associated with each protein-encoding gene in the SEED database. The main such characteristic was the presence of ‘functional coupling’ or ‘conserved gene clustering’ [24-27] for a prokaryotic member of the family, but other criteria were also computed when available as detailed below.

#### Detecting and analyzing gene clustering

Physical gene clustering is the tendency of functionally associated genes to be located near each other on the chromosome. Although not entirely absent in eukaryotes [28], such clustering is far more marked in prokaryotes, in which functionally related genes are often arranged in operons [29] or divergently transcribed from the same promoter region [24], or are simply neighbours or near-neighbours [24,30]. On average, ~35% of bacterial metabolic genes are in clusters [24]. A key point is that the more taxonomically diverse the genomes in which a cluster occurs, the more informative the cluster becomes [30]. A single gene family can be involved in different clusters in different taxa (all potentially diagnostic of its function), even if it is not clustered with informative genes in all taxa.

Several software tools in SEED that take advantage of gene clustering were used to select promising candidates, as well as to link unknown gene families to general metabolic pathways and to generate specific functional predictions during the next phase of the project:

(i) Strength of ‘functional coupling’ (FC) – measures the number of distantly related organisms (with 95% overall DNA sequence identity or less) in which two genes are located in each other’s vicinity. Close strains are not taken into account in this parameter, for example: all sequenced *Escherichia coli* genomes in SEED in which two particular genes are co-localized on the chromosome are counted as one when computing FC (see ref. [24] for a more formal treatment of this topic).

(ii) Length of cluster – reflects the number of genes involved in a specific cluster.

(iii) Evidence code ‘in cluster with non-hypothetical’ (cwn) – indicates that a gene family is functionally coupled to (tends to co-localize with) at least one other gene family that has been assigned a function that is considered ‘non-hypothetical’. The functional coupling score must be five or more for this code to apply.

(iv) Evidence code ‘in cluster with hypothetical’ (cwh) – as above, except it labels gene families that tend to co-localize with at least one other hypothetical gene family;

(v) Association of a gene family with a ‘clustering based’ subsystem in SEED (CBSS). These subsystems group hypothetical protein families solely on the grounds of co-localization patterns conserved across multiple genomes; however, manual subsystem encoding takes automatically pre-computed leads (such as evidence codes) to the next level. For example, comprehensive phyletic spread is determined for protein families that might have been labeled as ‘in cluster with hypothetical’ in merely a fraction of genomes. CBSS subsystems provided a useful starting point for the next phase of *in silico* analysis.

#### Other filtering factors

Other factors that were considered included:

(i) Phyletic spread – the number of distinct microbial species that harbored members of each hypothetical family under consideration, whether or not they were functionally coupled (see above). Widely distributed families were preferred over narrowly distributed ones.

(ii) Whether or not well studied model organisms such as *E. coli*, *Bacillus subtilis*, *Pseudomonas aeruginosa*, cyanobacteria, or yeast contained a member of the gene family in question, indicating likely availability of functional genomics data (e.g. expression arrays, gene essentiality data, protein interaction datasets) that could provide clues linking candidate families to general metabolic areas and aid specific functional predictions.

Comprehensive tables summarizing all types of association evidence (available as Additional Files 1 and 2) were used for manual sorting and evaluation to prioritize hypothetical plant/prokaryote gene families and to select candidates for further detailed *in silico* analysis.

### Linking unknown gene families to general metabolic areas

Our first goal was to link the prioritized gene families to a particular metabolic or functional area. For this, we built on the gene clustering associations captured as described above, using these to construct a corresponding experimental subsystem in the SEED database for each family (see Methods). Each such subsystem included all members of the focus gene family across all genomes available in the database, as well as gene families potentially associated with it (as implicated by gene clustering in at least a fraction of prokaryote genomes). Such integration of biological functions with genome sequences provided by subsystems allowed us to discard or strengthen the clustering associations and to evaluate the phylogenetic co-distribution between the gene family of interest and the associated families. We then further explored the associations by extending the analysis to Arabidopsis. Indeed, organization of genomic data in subsystems allows accurate extrapolation of functional associations between genes detected in microbial genomes onto other prokaryotes and even eukaryotes (e.g. Arabidopsis), albeit with caution. For example, in our study the degree of similarity between an Arabidopsis gene and its nearest prokaryotic homolog involved in gene clustering played an important role in evaluating the validity of such cross-kingdom projections. Additional ‘checks and balances’ were used in projecting functional leads and hypotheses developed via comparative analysis of prokaryotic genomes back to plant genes, which might not have preserved the function of their prokaryotic counterparts. For example, when linking unknown gene families to general metabolic areas, or to individual protein families via gene clustering, the validity of such associations in the context of plant physiology and biochemistry was considered, as well as its correspondence to Arabidopsis expression array data [31-33], protein localization [34,35], mutant phenotypes, and other functional genomics data (as illustrated in the case studies below). This first analysis yielded a list of 78 gene families linked to diverse metabolic areas (Additional file 2), including fatty acids, terpenes, vitamins, aromatic compounds, and sulfur, as well as iron-sulfur cluster assembly, oxidative damage protection, glutathione S-transferase-dependent detoxification, DNA repair, plastid/cell division, signalling systems, and metal homoeostasis. However, a clear bias reflecting the investigators’ areas of expertise was observed, with some ten in vitamin/cofactor metabolism and another fourteen in tRNA/RNA modifications. This emphasizes the value of combining multiple types of expertise to accurately predict and validate gene function.

### Predicting and testing precise molecular and biological functions

#### General strategy

The next step in the pipeline was creating a functional hypothesis that could be tested by genetic and/or biochemical experiments. This is by far the most labor intensive and intellectually challenging step in the pipeline. Multiple types of data need to be queried and integrated with biochemical insights in order to make reasonable and testable predictions. Clues can come from high-throughput data (protein complexes, phenotypes, microarrays) from any organism, from the literature (where data may be buried in supplemental tables and contain no reference to the gene family), or from analysis of the structure of a member of the family (e.g. from a structural genomics effort). Biochemical insight can come from cataloguing globally or locally missing genes, i.e. those that encode enzymes for which a gene has never been identified in any species or is absent in certain species [36-38].

An example of globally missing gene identified in this work is the Sua5/YrdC family involved in the universal carbamoylthreonyladenosine (t^6^A) modification in tRNA (case number 5 in Table 2 and summarized below). An example of a locally missing gene is the PTPS-III family (case number 4 in Table 2) that replaces the folate biosynthesis enzyme FolB in many bacteria and certain eukaryotes [39,40]. Biochemical insights can also come from noting the presence of two gene families annotated as fulfilling the same role, suggesting a possible duplication followed by functional divergence [41]. One such example is the COG0354 family, previously annotated in many genomes as the folate-dependent glycine cleavage system T protein (GcvT), but in reality a protein involved in the repair of iron-sulfur clusters (case number 1 in Table 2 and summarized below). For some families very precise functions could be predicted, e.g. methylation of a specific position in ribosomal RNA (At4g28830, case number 31 in Table 3) whereas for others the prediction remained more general but testable nonetheless. For instance, we were able to link certain members of the COG0523 family (case number 7 in table 2) to zinc homeostasis [42] and this general prediction was borne out by demonstrating that some members of the family have a role in survival in low zinc conditions ([43] and C. Blaby-Haas and V. de Crécy-Lagard, unpublished results). We were able to make testable functional predictions for 41 families (Additional file 2); the rationales for these predictions are summarized in the subsystem notes for each family in the SEED database. Table 2 lists the 19 families for which the prediction has been experimentally confirmed by us or others. Table 3 lists four families that were experimentally invalidated and another eight for which experiments are well advanced, for a total of 31 families. Three illustrative examples of validated predictions are described briefly below. The first and second of these are fully described elsewhere [44,45].

**Table 2:** Status of the experimentally validated families: cases 1-9 verified by us; cases 10-19 verified by others.

Case no.	TAIR ID	COG number/ gene name	Subsystem in SEED	Working functional prediction	Experimental verification status	Homologs annotated	Reference
1	At4g12130 At1g60990	0354 ygfZ	YgfZ	Folate-dependent protein for Fe/S cluster synthesis/repair in oxidative stress	Validated in *E. coli*, *Bartonella henselae*, *Haloferax volcanii*, *Arabidopsis*, *Leishmania*, yeast, mouse	327	[44] (2010)
2	At2g20830	3643	Experimental-histidine degradation	Alternative to 5-FCL (EC 6.3.3.2) as a way to metabolize 5-formyltetrahydrofolate	Verified in 5 prokaryotes	65	[45] (2010)
3	At1g29810 At5g51110	2154 phhB	Pterin carbinolamine dehydratase	Pterin-4-alpha-carbinolamine dehydratase (EC 4.2.1.96) with a role in Moco metabolism	Validated in 7 eukaryotes and 8 prokaryotes	217	[81] (2008)
4	none	0720	Experimental-PTPS	Replacement for FolB (EC 4.1.2.25)	Validated in 1 eukaryote and 8 prokaryotes	65	[40] (2009); [39] (2008)
5	At5g60590	0009 yrdC	YrdC-YciO-Sua5 protein family	Required for threonylcarbamoyl-adenosine (t(6)A) formation in tRNA	Validated in yeast, archaea and 2 bacteria. Arabidopsis in progress.	745	[60] (2009)
6	At2g45270 At4g22720	0533 ygjD	YrdC-YciO-Sua5 protein family	Required for threonylcarbamoyl-adenosine (t(6)A) formation in tRNA	Validated in yeast	691	[82] (2011)
7	At1g15730 At1g26520 At1g80480	0523	COG0523	Diverse metal chaperones	Validated in several bacteria	718	[42] (2009)
8	At3g13050	MFS superfamily NiaP homolog	Niacin-choline transport and metabolism	Niacin and/or choline transporter	Niacin but not choline transport shown for 3 bacterial proteins and the mouse protein Arabidopsis protein in progress.	133	Manuscript in prep
9	At1g76730	0212	5-FCL-like protein	Not a 5-FCL enzyme; involved in thiamine salvage	Cannot replace 5-FCL and lacks detectable 5-FCL activity	41	Manuscript submitted
10	At4g36400	0277 bll2569	COG0277	D-2-hydroxyglutarate dehydrogenase	D-2-hydroxyglutarate dehydrogenase	158	[83] (2009)
11	At5g10910	0275 mraW	16S rRNA modification within P site of ribosome	SAM-dependent methyltransferase involved in a process common to eubacteria and chloroplasts	16S rRNA m(4) C1402 methyltransferase (modification within P site of ribosome)	877	[84] (2010)
12	At1g45110	0313	16S rRNA modification within P site of ribosome	Tetrapyrrole family methyltransferase involved in a process common to eubacteria, chloroplasts, and possibly mitochondria	16S rRNA 2'-O-ribose C1402 methyltransferase (modification within P site of ribosome)	836	[84] (2010)
13	At5g18570 At1g07620	0536	Iojap	At5g18570 predicted to be plastidial, At1g07615 mitochondrial. Association evidence connects At5g18570 with plastidial iojap (At3g12930)	Essential for embryo development but specific function unclear	721	[85] (2009)
14	At1g49350	2313 yeiN	Pseudouridine catabolism	Sugar catabolism	Involved in pseudouridine metabolism in uropathogenic *E. coli*	108	In EC: [86] (2008)
15	At1g50510	0524 yeiC	Pseudouridine catabolism	Sugar catabolism	Involved in pseudouridine metabolism in uropathogenic *E. coli*	108	In EC: [86] (2008)
16	At4g10620 At3g57180 At3g47450	1161 yqeH	At4g10620 At3g57180 At3g47450	GTP-binding protein YqeH, involved in replication initiation	At3g57180 (BPG2) functions in brassinosteroid-mediated post-transcriptional accumulation of chloroplast rRNA. At3g47450 (AtNOA1) is a GTPase that regulates nucleic acid recognition	180	[87] (2010) [88] (2008)
17	At3g24430 At4g19540 At5g50960	2151 apbC	Scaffold proteins for [4Fe-4S] cluster assembly (MRP family)	Fe-S cluster assembly proteins. The DUF59 (PaaD-like) domain of At3g24430 and its prokaryotic counterparts are also predicted to function in Fe-S cluster assembly.	At5g50960 (Nbp35) functions in Fe-S cluster assembly as a bifunctional molecular scaffold At3g24430 acts as a scaffold protein for [4Fe-4S] cluster assembly in chloroplasts	276	[89] (2009) [90] (2005)
18	At3g57000	1756	rRNA modification Archaea; rRNA methylation in clusters	rRNA modification enzyme	The *Methanocaldococcus jannaschii* ortholog is a pseudouridine-N1-specific methyltransferase.	31	[91] (2010)
19	At5g12040	0388	Omega-amidase	Omega amidase in methionine salvage pathway	Biochemical characterization of the rat and mouse orthologs	113	[92] (2009) [93] (2009)

**Table 3:** Status of the families invalidated by us (cases 20-23) or in progress (cases 24-31).

Case no.	TAIR ID	COG number/ gene name	Subsystem in SEED	Working functional prediction	Experimental verification status	Homologs annotated	
20	At5g43600	0624	Experimental - Histidine Degradation	Alternative form of N-formylglutamate deformylase (EC 3.5.1.68)	No deformylase actiivity detected in *Streptomyces avermitilis* protein	*24*^a^	
21	At2g23390	3146	COG3146	Pterin-dependent enzyme	*Xanthomonas campestris* protein lacks benzoate hydroxylase activity in complementation assay	*236*	
22	At2g04900	2363 ywdK	COG2363	Thiamine-related transporter	*E. coli* protein does not mediate uptake of thiazole or hydroxymethylpyrminidine	*221*	
23	At1g09150	2016	rRNA modification Archaea; DOE-COG2016	Ribosome assembly/translation termination	In progress in yeast and *H. volcanii.* Hypothesis that it is involved in acp3psi synthesis **invalidated by Fournier lab^b^**	*30*	
24	At4g26860 At1g11930	0325 yggS	PROSC	Pyridoxal phosphate enzyme related to glutamate metabolism	In progress in *E. coli*	*589*	
25	At1g78620 At5g19930	1836 alr1612	COG1836	Phytol-phosphate metabolism	Shown to be an essential gene in *Synechocystis* 6803. Further work in progress in Arabidopsis	*77*	
26	At5g12950 At5g12960	3533 SAV1144	DOE COG3533	Hydroxyproline-galactosyl hydrolase	In progress in *X. campestris*	*82*	
27	At3g09250	4319 gll0142	COG4319	Folate or pterin metabolism enzyme, possibly an alternative DHFR (EC 1.5.1.3), a pterin reductase, or a dihydroneopterin triphosphate hydrolase	*Streptomyces coelicolor*, Arabidopsis At3g09250, and *Nostoc punctiforme* proteins failed to complement *E. coli* folA (DHFR) strains	*59*	
28	At3g12930 At1g67620	0799 alr4169	Iojap	NAD-dependent ribosomal modification, possibly involving phosphoester hydrolysis	No pyrophosphatase or NAD cleavage activity detected in *E. coli* YbeB or NadD-YbeB fusion protein from *Wolinella succinogenes*	*672*	
29	At3g01920	0009 yciO	YrdC-YciO-Sua5 protein family	RNA/protein modification	In progress in *E. coli*	*195*	
30	At1g03030	1072 yggC	Experimental-yggC	Sugar/polyol kinase	In progress in *E. coli*	*48*	
31	At4g28830	2263	rRNA modification Archaea	Predicted RNA methylase COG2263	In progress in *H. volcanii*	*49*	

^a^ Numbers in italics are for members of families for which the prediction has been invalidated or is in progress, they have not been included in the final count.

^b^ S. Fournier and W. Decatur, University of Massachusetts (unpublished)

#### COG0354 (At4g12130, At1g60990)

**Figure 2:**
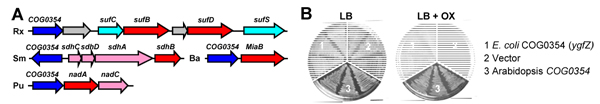
**Clustering arrangements of genes encoding COG0354 and functional complementation of an *E. coli* COG0354 deletant by an Arabidopsis COG0354.** (A) Clustering of COG0354 genes with Fe/S-related genes. Blue, COG0354; red, Fe/S proteins; rose, proteins in same complex or pathway as Fe/S proteins; turquoise, Fe/S cluster assembly proteins. Rx, *Rubrobacter xylanophilus*; Sm, *Stenotrophomonas maltophilia*; Pu, *Pelagibacter ubique*. **(B)** Growth of an *E. coli* COG0354 (*ygfZ*) deletant harboring plasmid-borne *E. coli ygfZ*, Arabidopsis mitochondrial COG0354, or vector alone on LB medium or LB plus the oxidative stress agent plumbagin (OX) (30 μM), arabinose (0.02% w/v), and appropriate antibiotics.

Bacterial genes encoding COG0354 (case number 1 in Table 2), the GcvT paralog noted above, often cluster with diverse iron/sulfur (Fe/S) proteins (shown in red in Fig. 2A), and proteomic data [46,47] show induction by oxidative stress and confirm an Fe/S association. Moreover, the COG0354 protein is required for full activity of certain Fe/S enzymes in *E. coli*[48] and yeast [49]. We therefore predicted that COG0354 is a folate-dependent enzyme (based on its homology to the folate-dependent protein GcvT) involved in assembly or repair of Fe/S proteins, particularly under oxidative stress. Consistent with this prediction, deleting the gene encoding COG0354 in *E. coli* (*ygfZ*) increased oxidative stress sensitivity, and the stress-sensitive phenotype was complemented by expressing a plant COG0354 protein (Fig. 2B). Folate-dependence was established by using NMR to demonstrate stereoselective folate binding by recombinant *E. coli* COG0354, and by showing that *in vivo* activity of the *E. coli* Fe/S protein MiaB is as seriously impaired by deleting the folate synthesis gene *folE* (which eliminates folates) as by deleting *ygfZ*, i.e. removing folates had the same impact as removing COG0354 [44].

#### COG3643 (At2g20830)

**Figure 3:**
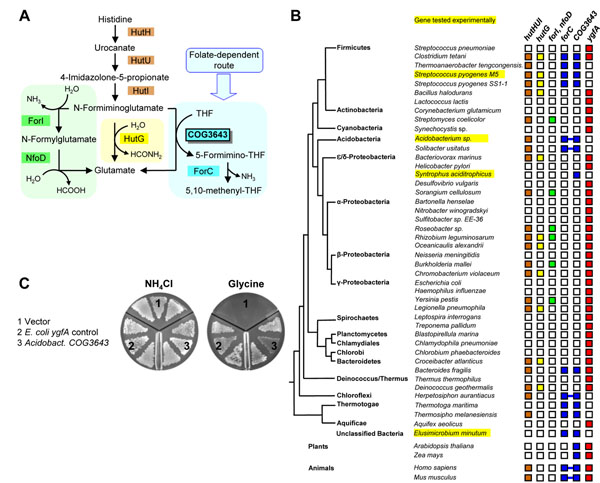
**COG3643 in relation to the Hut pathway. (A)** Hut pathway; note the three different routes. **(B)** The distribution of histidine utilization genes among bacterial and eukaryal genomes in relation to that of the *ygfA* gene for 5-formyltetrahydrofolate disposal. Gene colors correspond to different parts of the pathway as in part A. Lines between boxes denote gene fusions. **(C)** Growth of an *E. coli ygfA* deletant harboring plasmid-borne *E. coli ygfA*, *Acidobacterium* COG3643, or vector alone on minimal medium with NH_4_Cl or glycine as sole nitrogen source. The medium contained 1 mM IPTG and appropriate antibiotics.

The histidine utilization (Hut) pathway occurs in certain bacteria and animals, but not plants. The Hut pathway up to the intermediate *N*-formiminoglutamate is invariant, but thereafter there are three routes to the end-product glutamate, one of which involves a formiminotransferase, COG3643 (Fig. 3A). Comparative genomics analysis showed that bacteria that have a formiminotransferase-type Hut pathway generally lack the *ygfA* gene encoding 5-formyltetrahydrofolate cycloligase, the key enzyme required to recycle 5-formyltetrahydrofolate, which inhibits various folate-dependent enzymes and is formed by a side reaction of serine hydroxymethyltransferase in the presence of glycine (Fig. 3B). This striking observation led us to predict that formiminotransferase or formiminotransferase paralogs can replace YgfA. This prediction fits with classical biochemical data showing that mammalian formiminotransferase can mediate formyl transfer from 5-formyltetrahydrofolate to glutamate, albeit at a low rate [50,51]. The prediction was supported by showing that various prokaryotic COG3643 genes (highlighted in Fig. 3B) complement the growth phenotype of an *E. coli ygfA* deletion mutant (which cannot use glycine as sole nitrogen source, presumably because 5-formyltetrahydrofolate accumulation inhibits the folate-dependent glycine cleavage reaction). Representative data for the *Acidobacterium* COG3643 gene are shown in Fig. 3C. Folate analysis of the *ygfA* deletant with and without complementing COG3643 genes confirmed that the deletant accumulated 5-formyltetrahydrofolate and that COG3643 genes reversed this accumulation [45]. Furthermore, characterization of recombinant COG3643 proteins showed their kinetic characteristics to be consistent with an *in vivo* role in 5-formyltetrahydrofolate recycling [45]; this biochemical corroboration is important since functions carried out by ectopically overexpressed genes do not necessarily reflect their native function. Taken together, this evidence suggests that COG3643 paralogs in plants may likewise replace YgfA. Consistent with this possibility, the *ygfA* knockout in Arabidopsis has a mild phenotype, pointing to the existence of an alternative route for disposal of 5-formyltetrahydrofolate [52].

#### YrdC/Sua5 (At5g60590)

**Figure 4:**
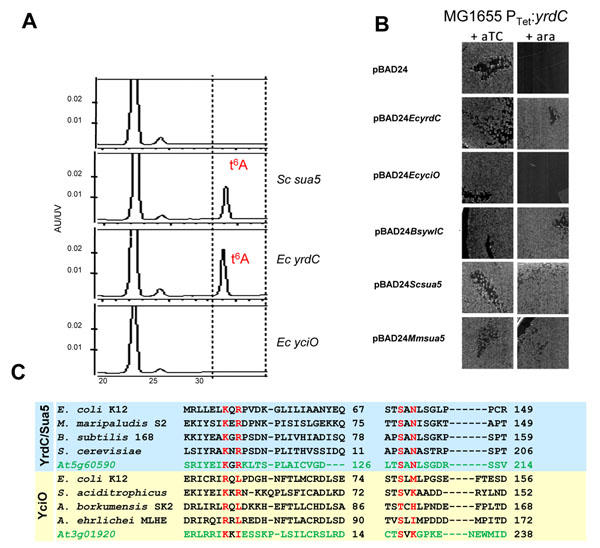
**Separation of the COG009 family into two subgroups YrdC and YciO based on motifs and functional assays. (A)** Complementation of the t^6^A^-^ phenotype of the yeast Δ*sua5* (YGN63) strain by the *E. coli yrdC* gene but not the *E. coli yciO* gene. **(B)** Complementation of the *yrdC* essentiality phenotype in *E. coli* by *yrdC* subfamily members from *E .coli* (*EcyrdC*), *Bacillus subtilis* (*BsywlC*), *Methanococcus maripaludis* (*MmyrdC*) and yeast (*Scsua5*) but not by *yciO* from *E. coli* (*EcyciO*). All genes were cloned in pBAD24 [95] and were therefore expressed in the presence of arabinose (Ara, 0.2%) and transformed in an *E.coli* strain carrying the chromosomal copy of *yrdC* under P_Tet_ control [96] that does not grow in the absence of anhydrotetracycline (ATc, 50 ng/ml). **(C)** Signature motif of the functional homologs of YrdC (KxR/SxN) that are not found in the YciO subfamily. In green are the two homologs from Arabidopsis and their distribution.

The universal base modification t^6^A occurs at position 37 in a subset of tRNAs decoding ANN codons. The biogenesis of this complex modification is yet to be elucidated but is known to require threonine, ATP, and bicarbonate [53-55]. COG0009 was predicted as a possible candidate for a missing t^6^A biosynthesis family because it occurs in all genomes sequenced to date, is known to bind double-stranded RNA [56], and has been linked to defects in translation in both prokaryotes and eukaryotes [57,58]. This conjecture was supported by sequence homology with the [Ni-Fe] hydrogenase maturation protein HypF, which catalyzes a reaction analogous to the one expected for a t^6^A enzyme [59]. The COG0009 family can be further split based on sequence comparison into three subfamilies: YrdC, Sua5 (YrdC with an extra domain termed Sua5), and YciO. One or two members of this family are present in each genome; for example, the Arabidopsis and *E. coli* genomes contain two, YrdC (At5g60590) and YciO (At3g01920), while the *Saccharomyces cerevisiae* genome contains only one, Sua5 [60]. We showed that (i) tRNAs from *S. cerevisiae* strains lacking *sua5* do not contain t^6^A and that this phenotype is complemented by transforming with a plasmid encoding the wild type gene (Fig. 4A); (ii) the homologs from *B. subtilis*, *M. maripaludis*, *E. coli yrdC*, but not *E. coli yciO* also complement the phenotype; (iii) the *yrdC* homolog is essential in *E. coli*, whereas *yciO* is not; (iv) *S. cerevisiae*, *B. subtilis*, *M. maripaludis yrdC* genes but not *E. coli yciO* are able to complement the lethality phenotype of *yrdC* in *E coli* (Fig. 4B); and (v) *E. coli yrdC* is able to bind t^6^A apomodified tRNA^Thr^ but not unmodified transcript [60]. Therefore, members of the YrdC/Sua5 family are involved in t^6^A biosynthesis. In Arabidopsis, At5g60590 and At3g01920 are annotated as related to the YrdC family. However, based on comparative sequence analysis combined with genetic orthologous complementation tests these can be distinguished. As depicted in Fig. 4C, the YrdC family is characterized by the KxR/G …SxN signature sequence; At5g60590 is therefore most probably part of the YrdC family while At3g01920 is not.

### Propagating validated and predicted gene functions to other organisms

Subsystem-based organization of genomic data in SEED implies delineation and maintenance of isofunctional gene groups [61]. This strategy greatly facilitates not only the development of functional predictions for uncharacterized genes, but also accurate projection of this knowledge, once verified, to other species. For example, annotations for the 19 families developed in the course of this study and experimentally confirmed by us or others have been propagated to a total of 6,297 genes in some 900 complete or nearly complete bacterial, archaeal, and eukaryotic genomes currently available in the SEED database (Table 2). Furthermore, we believe there is merit in accurate and exhaustive propagation of yet untested predictions to all orthologs in all available genomes, early in the process of *in silico* analysis. This allows complete and accurate cataloguing of functional homologs for each gene family under study, thus revealing its phyletic spread, co-occurrence with known gene families, potential associations with specific features of an environmental niche – all of which can serve as additional clues for developing specific functional hypotheses. For this reason we have built subsystems in the public SEED database for 78 families (Additional file 2) so that readers can produce and test their own predictions for gene families that fall in their area of expertise. The total number of annotated genes in these families exceeds 22,000.

### Comparing results to those from automated functional prediction platforms

Several recently developed platforms seek to automatically integrate comparative genomics, high-throughput experimental data, and literature reports to make gene function predictions [46,62]. For ten gene families that were predicted and validated, we analyzed the accuracy of the corresponding predictions from the two most relevant predictions platforms, eNet (*E. coli*) [46] and AraNet (Arabidopsis) [62] (Table 4). The predictions from these automated platforms were mostly wrong. At best, they produced a general annotation that was in the right functional area such as ‘folate dependent regulatory protein’ by eNet and ‘iron-sulfur assembly protein’ by AraNet for the COG0354 family. However, both of these came straight from the literature, not from associations, and can thus hardly be called predictions; furthermore, these two correct ‘predictions’ were buried in long lists of incorrect ones. Thus, whenever a prediction was possible, the automated platforms failed to make a correct and precise one.

**Table 4:** Comparison of functional predictions in eNet and AraNet for 10 of the protein families.

TAIR ID	E.coli ortholog	Working functional prediction	eNet predictions	AraNet predictions^a^
**At4g12130,** At1g60990	YgfZ	Folate-dependent protein for Fe/S cluster synthesis/repair in oxidative stress	**Annotation based on**[ 94]**:** Predicted folate-dependent regulatory protein. **Prediction:** Energy production and conversion, ion transport	**For At4g12130:** NAD biosynthesis (2.96), electron transport, cellular respiration, N-terminal protein amino acid modification, miRNA-mediated gene silencing, production of miRNAs, methylglyoxal catabolic process to D-lactate, embryonic development, etc
At2g20830	none	Alternative to 5-FCL (EC 6.3.3.2) as a way to metabolize 5-formyltetrahydrofolate	n/a	Response to wounding (1.86), defense response, response to oxidative stress, phenylpropanoid biosynthesis, response to other organism, boron transport, glucosinolate biosynthesis (0.89)
At1g29810, At5g51110	none	Pterin-4-alpha-carbinolamine dehydratase (EC 4.2.1.96) with a role in Moco metabolism	n/a	**For At1g29810:** electron transport (3.13); carotenoid biosynthesis (2.29); brassinosteroid biosynthesis (2.16); fatty acid metabolic process (2.06); photosynthesis, light reaction (1.99); sulfate assimilation (1.98); lignin biosynthesis (1.87)
AT5g12040	YafV	Omega amidase in methionine salvage pathway	Predicted C-N hydrolase family amidase, NAD(P)-binding	indoleacetic acid biosynthesis (4.27), cellular response to sulfate starvation, cyanide metabolic process, glucosinolate catabolic process, detoxification of nitrogen compound, methylglyoxal catabolic process to D-lactate (1.59)
At5g60590	YrdC	Required for threonylcarbamoyladenosine (t(6)A) formation in tRNA	**Annotation based on**[ 57]: Predicted ribosome maturation factor. **NO prediction**	rRNA processing (3.88), dATP biosynthesis from ADP, histidine biosynthesis, mitochondrial ATP synthesis coupled proton transport, cellular respiration, ATP synthesis coupled proton transport, regulation of transcription (2.07)
At2g45270, At4g22720	YgjD	Required for threonylcarbamoyladenosine (t(6)A) formation in tRNA	**Prediction:** Predicted peptidase (Amino acid transport and metabolism)	**For At2g45270:** transcription initiation (6.19), positive regulation of transcription, chlorophyll biosynthesis, porphyrin biosynthesis, phospholipid biosynthesis, electron transport, ATP-dependent proteolysis, N-terminal protein amino acid modification (1.81)
At1g15730, At1g26520, At1g80480	YjiA YeiR	Metal chaperone-Zinc homeostasis	**Prediction for b4352:** Inorganic ion transport and metabolism, response to stress; **Prediction for b2173:** Lipid transport and metabolism, RNA related, Regulation of transcription DNA dependent	**For At1g15730:** nitrogen compound metabolic process (4.04); positive regulation of metalloenzyme activity (4.04)
At1g76730	none	Not a 5-FCL enzyme; involved in thiamine salvage	n/a	Tetrahydrofolate metabolic process (4.56), negative regulation of transcription, response to abscisic acid stimulus (0.89)
At4g36400	none	D-2-hydroxyglutarate dehydrogenase	n/a	Cytoskeleton organization and biogenesis (2.39), actin cytoskeleton organization and biogenesis, ubiquitin-dependent protein catabolic process, response to light stimulus, response to wounding, seed germination (1.24)
At1g45110	yraL	Tetrapyrrole family methyltransferase involved in a process common to eubacteria, chloroplasts, and possibly mitochondria	**Prediction:** Replication, recombination and repair; RNA related, Translation	Toxin catabolic process (5.49), response to oxidative stress, cellular response to water deprivation, response to jasmonic acid stimulus, response to ozone, isoprenoid biosynthesis, electron transport (1.45)

^a^ Only a few top predictions (out of 30 routinely returned) for AraNet are shown. They are sorted by the AraNet score estimating the gene’s association with each particular process (given in brackets for the first and last predictions shown here).

## Conclusions

The analysis presented here shows that combining comparative genomics with expert intellectual input enabled correct functional annotation of 19 gene families by only a few researchers, in a short time (three years), and at a moderate cost (<$1M). This number of successful functional predictions is roughly comparable to the number made through the entire structural genomics effort [63], involving many more people, a much longer period, and far greater expense. The cost-effectiveness of our approach is thus perhaps its most striking feature.

Our analysis also underscores the imperative of combining molecular function and biological context to annotate function as shown in the COG3643 example. Homology and even *in vitro* assays would have labeled this family – correctly, but incompletely and misleadingly – as a formiminotransferase. Only by interpreting phylogenetic distribution data with biochemical insight and then applying complementation tests was this family correctly annotated as an alternative to 5-formyltetrahydrofolate cycloligase to metabolize 5-formyltetrahydrofolate. It is noteworthy that in this and most of our other successful predictions, there is a strong bias towards the authors’ areas of expertise – from which the obvious inference is that other experts would, equally easily, have been able to predict additional sets of functions. For this reason we have built subsystems in the public SEED database for 78 families and made available the raw comparative genomic data for all gene families shared between Arabidopsis and prokaryotes (Additional file 1A, 1B, and 1C) so that other experts can bring their insight to our analysis in order to make and test their own predictions.

Finally, the only plant genomes available when this effort started were Arabidopsis, rice, and poplar, and rich post-genomic resources were available only for Arabidopsis. We accordingly focused our work on gene families common to Arabidopsis and prokaryotes. However, now that other plant genomes are pouring in (some 20 are available already and many more are in the pipeline) it is clear that almost all of the families we investigated have orthologs in other plants, making our work of immediate value in annotating other plant genomes. Furthermore, the rapid growth of microarray databases and other post-genomic resources for plants besides Arabidopsis (e.g. [64-66]) is providing many sources of association evidence to reinforce the approach that we have pioneered here.

## Methods

### Bioinformatics

The SEED genomic database and software suite [10], publicly available at http://theseed.uchicago.edu/ (see http://TheSEED.org for access to data relating to the SEED Project) was the main comparative genomics platform of this study. This database hosts all validated and proposed functional predictions developed in the course of this study for 78 genes families analysed in this study.

The SEED organizes genomic data in the form of subsystems (typically metabolic pathways or structural complexes) covering all organisms rather than on an organism-by-organism basis. Subsystems are developed and maintained by experts to capture the current status of knowledge of specific biological processes in model, well characterized organisms and to project this knowledge to other species via comparative genomics and metabolic reconstruction techniques [10]. Each subsystem includes a set of functionally related protein families (jointly encoding a specific pathway, process, or structural complex) across all available genomes (874 bacterial, 58 archaeal, and 29 eukaryotic complete and nearly complete genomic sequences as of July 2010). In SEED large homology-based protein families are broken into isofunctional subfamilies (‘functional roles’) based on genome context, functional context, phyletic profiling, shared regulatory sites, and other homology-independent clues. Association of each functional role with the corresponding subsystem(s) provides rich two-dimensional functional/phylogenetic context for each subfamily, leading to far more accurate annotations than the usual approach of annotating the genes within a single organism. Furthermore, the subsystem spreadsheet is used in SEED as a framework for integration of various types of functional data organized as *gene* attributes (e.g. gene clustering on a chromosome, expression array data, gene essentiality, etc.) and *organism* attributes (oxygen requirement, motility, pathogenicity, etc), which provide valuable non-homology based clues for functional predictions for uncharacterized genes.

All Subsystems created in this study, as well as over 1300 resident subsystems in SEED encoding all aspects of microbial physiology and metabolism are available on the public SEED server at http://theseed.uchicago.edu/FIG/SubsysEditor.cgi. They are regularly updated to accommodate newly sequenced bacterial genomes as well as novel experimental data and other relevant data as they become available.

Phylogenetic occurrence profiles were analysed using the Signature Genes tool on the NMPDR server (http://www.nmpdr.org/FIG/wiki/view.cgi/FIG/SigGenes). This tool identifies gene families that are common to a selected group of genomes, or those that differentiate one group of genomes from another. Annotations for paralog families were made using physical clustering when possible or by building phylogenetic trees using the ClustalW tool [67,68] integrated in SEED or deriving specific motifs.

We also used the bioinformatic tools and resources at NCBI (http://www.ncbi.nlm.nih.gov) and KEGG (http://www.genome.jp/kegg) [69], BRENDA (http://www.brenda-enzymes.info/) [70], PHYRE (Protein Homology/analogY Recognition Engine http://www.sbg.bio.ic.ac.uk/phyre/) [71], the Pfam database (http://pfam.sanger.ac.uk) [72], and specialized genomic resources and collections of functional genomic data for Arabidopsis, yeast, and various bacterial species, including: TAIR (The Arabidopsis Information Resource http://www.arabidopsis.org/) [21]; SGD (*Saccharomyces* Genome Database http://www.yeastgenome.org) [22]; MicrobesOnline (http://www.microbesonline.org/) [73]; EcoGene (http://ecogene.org/) [74]; EcoCyc (Encyclopedia of *E. coli* genes and metabolism (http://biocyc.org/ECOLI/) [75]; Cyanobase (http://genome.kazusa.or.jp/cyanobase) [76]; *Pseudomonas* genome database (http://www.pseudomonas.com/) [77]; and Rhodobase (http://rhodobase.org/index.php). Collections of Arabidopsis global expression and proteomics data with on-line tools for visualization and analysis: ATTED (http://www.atted.bio.titech.ac.jp/) [78]; Golm Transcriptome database (http://csbdb.mpimp-golm.mpg.de/csbdb/dbxp/ath/ath_xpmgq.html, [31-33]); PED, Plant Gene Expression Database (http://bioinfo.ucr.edu/projects/Unknowns/external/express.html); Genevestigator (https://www.genevestigator.com) [31-33]; PPDB, The Plant Proteome Data Base (http://ppdb.tc.cornell.edu/) [79]; PDB, The Protein Data Base (http://www.rcsb.org/pdb/home/home.do) [80].

### Experimental validations

Methods for the three experimental validation vignettes described above are already, or soon will be, described in the authors’ publications.

## Competing interests

The authors declare that they have no competing interests.

## Authors' contributions

V de C-L and ADH conceived the study and, with SG, carried out the bioinformatic and function prediction work, and drafted the manuscript. RO and AW participated in the bioinformatic analysis. BEY, MB, IKB, CH-B, LJ, AL-N, AP, and JCW performed the experimental validation experiments. BEY participated in manuscript preparation. All authors read and approved the final manuscript.

## Supplementary Material

Additional file 1 Gene families shared between plants and prokaryotes: unique Arabidopsis genes (1A), paralogous Arabidopsis gene families with 2 members (1B), paralogous Arabidopsis gene families with 3 members (1C). Click here for file

Additional file 2 Gene families shared between plants and Prokaryotes, that were linked to general areas of metabolism and physiology, or associated with more specific potential functions. All additional files table were also made available on: http://www.theseed.org/Papers/20101120/. Click here for file
